# Ultrasensitive and Rapid Detection of Procalcitonin via Waveguide-Enhanced Nanogold-Linked Immunosorbent Assay for Early Sepsis Diagnosis

**DOI:** 10.1021/acs.nanolett.3c04762

**Published:** 2024-01-22

**Authors:** Devesh Barshilia, Jhen-Jie Huang, Akhil Chandrakanth Komaram, Yi-Che Chen, Chun-Da Chen, Min-Yu Syu, Wen-Cheng Chao, Lai-Kwan Chau, Guo-En Chang

**Affiliations:** † Department of Mechanical Engineering, National Chung Cheng University, Chiayi 621301, Taiwan; ‡ Advanced Institute of Manufacturing with High-Tech Innovations (AIM-HI), National Chung Cheng University, Chiayi 621301, Taiwan; § Department of Chemistry and Biochemistry, National Chung Cheng University, Chiayi 621301, Taiwan; ∥ Department of Laboratory Medicine, National Taiwan University Hospital Yunlin Branch, Yunlin 640, Taiwan; ⊥ Department of Critical Care Medicine, Taichung Veterans General Hospital, Taichung 402202, Taiwan; f Center for Nano Bio-Detection, National Chung Cheng University, Chiayi 621301, Taiwan

**Keywords:** Planar waveguide, Particle plasmon resonance, Optical biosensor, Point of care, Procalcitonin, Sepsis

## Abstract

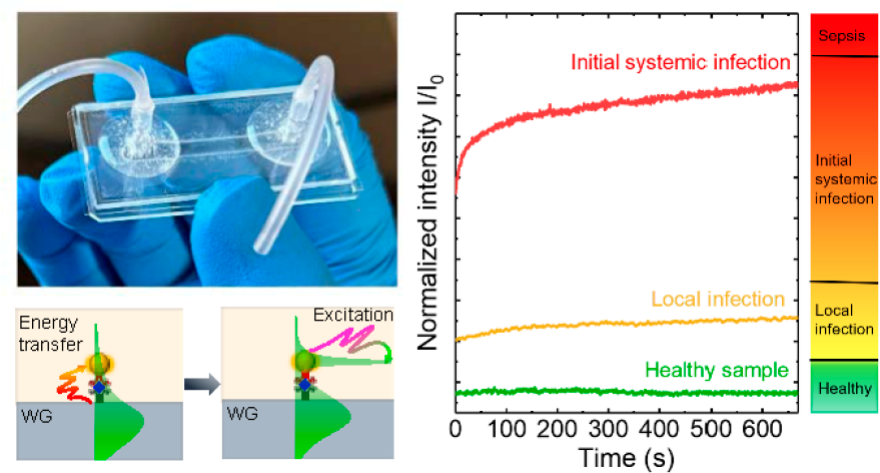

Sepsis, a life-threatening inflammatory response, demands economical, accurate, and rapid detection of biomarkers during the critical “golden hour” to reduce the patient mortality rate. Here, we demonstrate a cost-effective waveguide-enhanced nanogold-linked immunosorbent assay (WENLISA) based on nanoplasmonic waveguide biosensors for the rapid and sensitive detection of procalcitonin (PCT), a sepsis-related inflammatory biomarker. To enhance the limit of detection (LOD), we employed sandwich assays using immobilized capture antibodies and detection antibodies conjugated to gold nanoparticles to bind the target analyte, leading to a significant evanescent wave redistribution and strong nanoplasmonic absorption near the waveguide surface. Experimentally, we detected PCT for a wide linear response range of 0.1 pg/mL to 1 ng/mL with a record-low LOD of 48.7 fg/mL (3.74 fM) in 8 min. Furthermore, WENLISA has successfully identified PCT levels in the blood plasma of patients with sepsis and healthy individuals, offering a promising technology for early sepsis diagnosis.

Sepsis is a systemic inflammatory response caused by a bacterial infection that leads to the overproduction of cytokines, which can progress to organ system dysfunction, organ failure, and ultimately patient death. It is the most common cause of death worldwide.^1^ The progression rate of sepsis is rapid, and prompt intervention must be made within the “golden hour” of sepsis diagnosis.^2^ Early and proper treatment is extremely important to reduce the patient mortality rate during the early stages of sepsis.^3^ Currently, although some clinical methods to identify the onset of sepsis exist, detecting and completely quantifying inflammatory factors usually take 6–12 h (or more),^4^ which is significantly longer than the “golden hour”. This significantly delays proper treatment, thereby considerably lowering the patient survival rate. Therefore, rapid and reliable diagnostic methods are highly required. However, accurate and sensitive detection of sepsis is challenging, particularly in its early stages.^5^ Current clinical methods for sepsis diagnosis use blood samples obtained by the serial quantification of circulating biomarkers to monitor patients’ systemic responses for rapid patient stratification. Different biomarkers with different progress rates induced by sepsis have been investigated in practical applications.^6^ The rapid detection of key biomarkers can lead to useful diagnostic and prognostic outcomes.^7^ Currently, procalcitonin (PCT), C-reactive protein (CRP), and interleukin-6 (IL-6) are considered as effective biomarkers for sepsis diagnosis and prognosis.^2,8−10^ Among them, PCT is a popular biomarker whose elevated levels are considered as the clinical definition of sepsis. PCT has a broad biological range and short induction time after bacterial stimulus.^11^ In addition, high PCT levels can effectively distinguish sepsis from noninfectious systemic inflammatory response.^12^ Therefore, PCT is a very useful biomarker for diagnosing sepsis, predicting disease severity and outcomes and guiding antibiotic therapy. In a healthy body, PCT concentrations are <0.05 ng/mL.^12,13^ A PCT concentration between 0.5 and 2 ng/mL represents possible systemic infections, while PCT concentration between 2 and 10 ng/mL are suggestive of sepsis, and PCT concentrations of >10 ng/mL are indicative of severe sepsis or septic shock.^12,14^ Hence, highly sensitive and rapid biosensing techniques are required for PCT detection.

Current developments in bioanalytical technology and optical detection methods have led to new biosensing approaches that enable the sensitive, rapid, and cost-effective detection of meaningful biomarkers.^15,16^ Therefore, active research to develop optical biosensors for PCT including chemiluminescence,^17^ electrochemiluminescence,^18^ fluorescence,^19,20^ surface-enhanced Raman scattering,^21^ nanoplasmonics,^4,22^ surface plasmon resonance,^6^ and immunochromatographic assays is onging,^22,23^ owing to their unique advantages of low cost and specificity.^24^ However, many of these schemes do not meet the clinical sensitivity requirements for PCT detection due to the low molecular weight of about 13 kDa. In addition, bulky and expensive optical detection systems are typically required, which limits their practical applications. Recently, an optical biosensor based on a fiber optic nanogold-linked immunosorbent assay (FONLISA)^25^ with excellent sensitivity and limit of detection (LOD) for PCT was developed. However, the sensor fabrication process is complex, and the device is not mechanically robust. For successful commercialization, a highly sensitive, low-cost, robust, and rapid biosensor capable of quantitative measurements at ultralow PCT concentrations is highly desirable.

Here, we propose and develop waveguide-enhanced nanogold-linked immunosorbent assay (WENLISA) biosensors for sensitive and rapid PCT detection as a key enabler for early sepsis diagnosis. The WENLISA biosensor comprises a suspended glass planar WG on a glass substrate integrated with a microfluidic channel fabricated using simple, cost-effective, and vacuumless processes.^26^ In addition, to enhance the LOD to meet the clinical requirements for PCT detection, we introduced a new sensing strategy using gold nanoparticles (AuNPs) with a unique sandwich architecture. The AuNPs not only significantly modify the distribution of the evanescent wave of the guided mode within the WG but also induce strong resonance of AuNP plasmon modes to significantly enhance absorption near the PCT biomarker. Consequently, the concentration of the biomarker to be detected can be converted to the transmitted light intensity of the WG, which is modulated by the absorption of evanescent waves to achieve ultrasensitive and rapid PCT detection. The biosensing experiments revealed excellent PCT sensing performance with a record-low LOD of 48.7 fg/mL (3.74 fM), low nonspecific adsorption, and a short detection time of ∼8 min. Most importantly, we successfully verified the capability of WENLISA to accurately detect and quantify PCT levels in clinical samples by testing blood samples from both healthy individuals and patients with sepsis.

Figure 1a and Figure 1b present the 3D schematic and cross-section of the WENLISA biosensor, respectively. A suspended glass slab of thickness 200 μm is used as the slab WG on a glass substrate for the biosensor, wherein the air gap underneath the slab WG can significantly enhance the optical confinement for the guided modes owing to the enhanced contrast in the refractive index (RI). The glass WG is then integrated with a microfluidic module with channel dimensions as follows: *w* = 3 mm (width), *L* = 32 mm (length), *t* = 0.2 mm (height). The channel has an inlet and outlet at opposite ends for sample handling. The proposed WENLISA biosensors were fabricated without using any vacuum processes (as described in Supporting Information section S1), thus featuring low-cost and mass-production capabilities. An optical image of the fabricated WENLISA biosensor is shown in Figure 1c.

**Figure 1:**
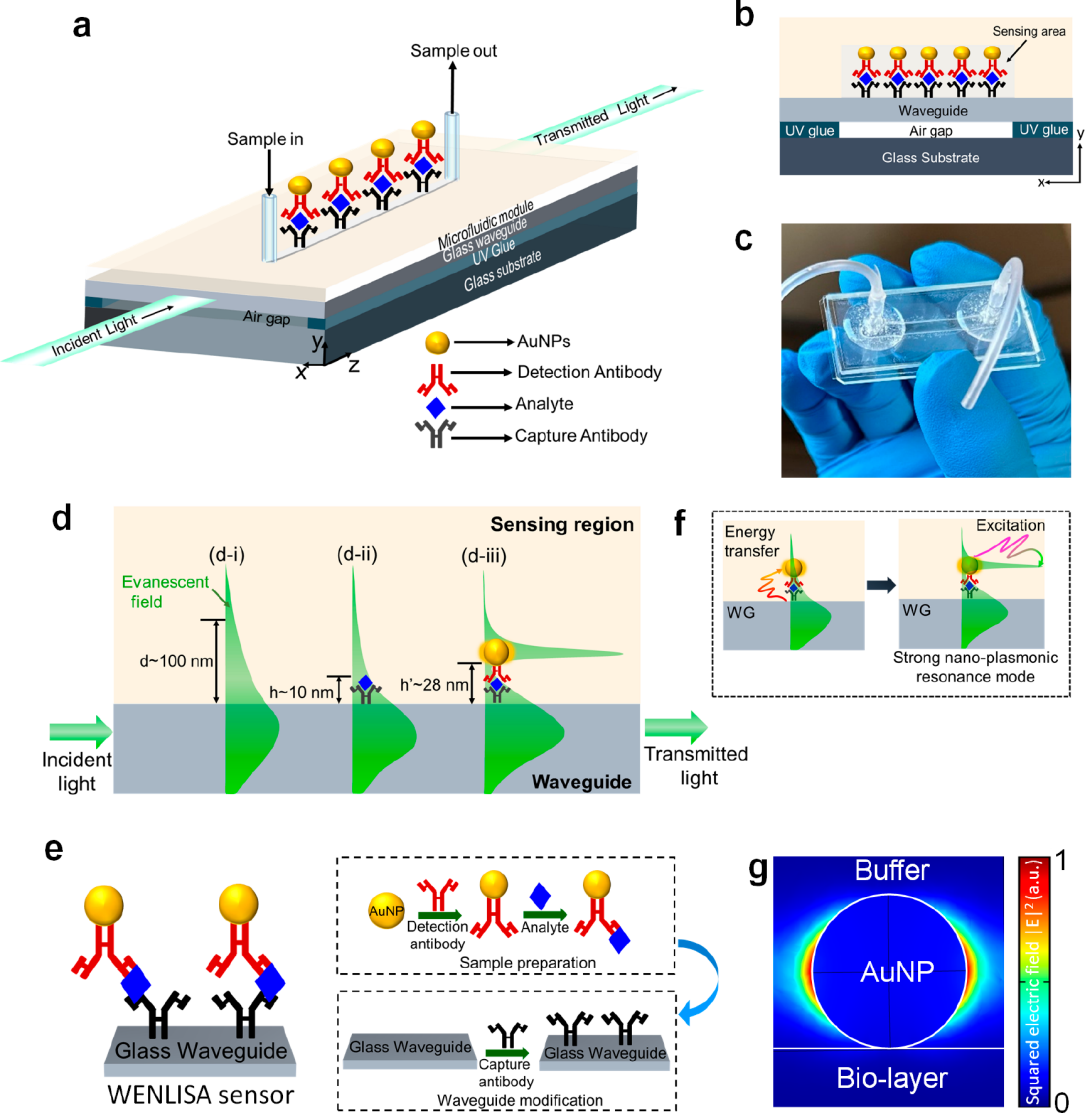
(a) 3D schematic diagram and (b) cross-section of the proposed WENLISA biosensor. (c) Optical image of the fabricated sensor. (d) Sensing mechanism of the WENLISA biosensor. (e) Process flow for the formation of the sandwich-like nanocomplex. (f) Schematic absorption and excitation process of nanoplasmonic resonance mode through AuNP. (g) Simulated squared electric field distribution of AuNP at λ = 521.5 nm.

For biodetection, the probe light from a low-cost and high-stability light-emitting diode (LED) is coupled to one end of the glass WG as shown in Figure S2 and then travels through the WG with an evanescent wave extending into the sensing region, as shown in Figure 1d. Details about the optical detection system are described in Supporting Information section S2. To enhance PCT detection sensitivity, the unique sandwich architecture adopted in our WENLISA sensor comprises two parts, as shown in Figure 1e. First, immobilized capture antibody molecules (Ab^C^) are modified on the WG surface. The AuNPs are then labeled with a detection antibody (AuNP@Ab^D^) and mixed with the target analyte, which are injected into the biosensor and captured by the Ab^C^. Figure 1d shows the sensing mechanism of the proposed WENLISA biosensor using the sandwich method. The simulated squared electric field distributions in the WG and its neighboring layers with and without AuNPs on the WG surface are described in Supporting Information section S3. When light travels through the WG, a significant evanescent wave extends to the sensing region, with a skin depth of approximately *d* ∼ 100 nm, as shown in Figure 1d–i. When biomolecules are attached to the WG surface, the evanescent waves interact with the analyte. However, when the analyte is small, as in PCT, a size mismatch between the skin depth of the evanescent wave and the length of the analyte results in very little change in the evanescent wave, as shown in Figure 1d-ii. This increases difficulty when detecting concentration using evanescent waves, and the sensitivity of this biosensor configuration is compromised. However, sandwiched nanocomplexes can dramatically improve this situation. When AuNPs are present, the evanescent wave of the probe light interacts with the AuNPs, resulting in an energy transfer to the AuNPs as shown in Figure 1f. As a result, the strong nanoplasmonic resonance induces a high electric field near the AuNP and significant optical absorption by the AuNP, as shown in Figure 1g. Additionally, nanoplasmonic resonance significantly modifies the distribution of evanescent waves, strongly reducing their skin depth and localizing it near the WG surface, as shown in Figure 1d-iii. Consequently, strong field enhancement and enhanced optical confinement promote strong light–matter interactions, inducing strong optical absorption of the guided wave. When more analyte molecules bound to AuNP@Ab^D^ are captured on the WG surface, the intensity of transmitted light (*I*(t)) decreases. Therefore, analyte concentration can be translated into transmitted light intensity to simultaneously achieve real-time and ultrasensitive biodetection.

Biodetection experiments were performed to assess the biosensing capabilities of WENLISA. Figure 2a depicts the background nonspecific adsorption test results using blank samples, an essential consideration in biosensors. To comprehensively evaluate nonspecific adsorption, we alternatively injected phosphate buffered saline (PBS) and AuNP@Ab^D^ solution into the WENLISA biosensor and synchronously recorded the real-time transmitted light intensity *I*(t). *I*_0_ is the average output light intensity when the microfluidic channel is initially filled with PBS. The normalized optical response (*I*(*t*)/*I*_0_) is shown in Figure 2a. Upon injecting the AuNP@Ab^D^ solution, a significant decrease in the transmitted light intensity was observed, which was attributed to a noticeable change in the RI of the solution and light scattering by the AuNPs in the solution. However, no biomolecular binding kinetic curve^27^ was detected because no biomolecular binding reactions occurred. Upon injection of the PBS solution, the transmitted light intensity reverted to approximately its original level. To facilitate quantitative analysis, a comparison was made between the average intensity obtained by injecting a sample comprising a AuNP@Ab^D^ solution and when reflushing it with PBS (*I*_n_). The normalized sensor response is defined as Δ*I*/*I*_0_, where Δ*I* = *I*_0_ – *I*_n_.^28^ From the nonspecific adsorption test results using blank solutions, we obtained the average nonspecific adsorption values Δ*I*/*I*_0_ = 0.000 82 ± 0.000 14 by serially injecting PBS, AuNP@Ab^D^, and PBS again in four different biosensor chips, and the background absorption level *B* = 0.0012 (Supporting Information section S4). The results highlight the very low nonspecific adsorption of the biosensor.

**Figure 2:**
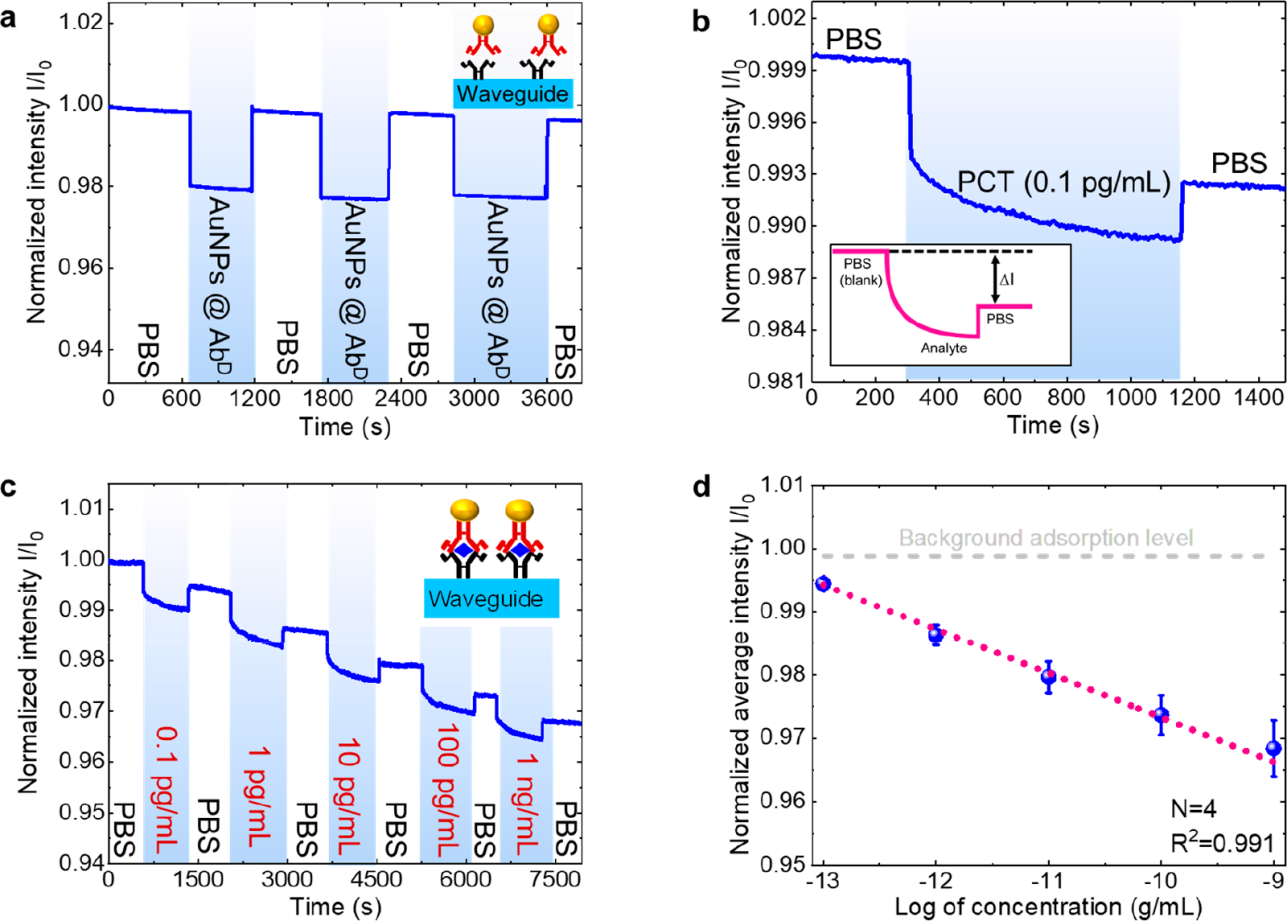
(a) Real-time response for the nonspecific adsorption experiment. (b) Real-time response for a sample with PCT concentration of 0.1 pg/mL. Inset: change in transmitted light intensity on analyte injection. (c) Real-time responses for serial sample injections at different PCT concentrations. (d) Calibration curve for normalized intensity as a function of PCT concentration. The gray dashed line represents the background adsorption level. The red dotted line represents the fit to the experimental data for the determination of limit of detection (LOD). Four experiments were conducted to determine the mean and the standard derivation as error bars.

Next, the biodetection results were evaluated by using PCT standard solutions. Figure 2b shows the optical response of our WENLISA biosensor with PBS as a blank solution, followed by the injection of a PCT standard solution of concentration 0.1 pg/mL and another injection of PBS. The solution flow was stopped after each injection. After a stable baseline was observed in the blank sample, a PCT standard solution was injected into the sensing region, and the transmitted light intensity suddenly dropped. This intensity drop is attributed to the RI change and the extinction of light by the AuNPs mainly through absorption as they have a large extinction coefficient of *k* = 2.2 at λ = 521.5 nm.^26^ Subsequently, the transmitted light intensity decreased exponentially. This is attributed to the selective capture of PCT molecules binding with AuNP@Ab^D^ to form AuNP@Ab^D^–analyte–Ab^C^ sandwich-like nanocomplexes on the WG surface, significantly inducing nanoplasmonic resonance and resulting in a gradual decrease in the transmitted light intensity over time.^14^ These results clearly demonstrate diffusion-limited molecular binding during PCT biodetection. Next, PBS was injected into the sensor to wash the unbound AuNP@Ab^D^ and eliminate light scattering and RI changes by AuNPs in the solution, leading to an increase in the transmitted light intensity. Consequently, the change in transmitted light intensity owing to capture of AuNP@Ab^D^–analyte by the immobilized Ab^C^ is determined to be Δ*I*, which is defined as the difference in average transmitted light intensity with injection of PBS solution before and after sample injection containing the analyte, as shown in the inset of Figure 2b. In addition, from the biomolecular binding kinetic curve, the response time of the biosensor was determined to be 10% of the initial value and 90% of the equilibrium value of the biomolecular binding kinetic curve,^29^ thereby yielding a response time of approximately 8 min, highlighting the rapid detection capability of our biosensor.

Figure 2c illustrates the optical response of the WENLISA biosensor with PCT standard samples over a wide range of concentrations from 0.1 pg/mL to 1 ng/mL using a single sensor. As the PCT concentration increased, the light intensity decreased significantly due to an increase in the number of AuNPs bound to the WG surface. Figure 2d shows the calibration curve of the normalized average intensity (Δ*I*/*I*_0_) as a function of the logarithmic value of the PCT concentration extracted from Figure 2c. Using the calibration curve, the system LOD was determined as follows:
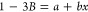
1
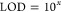
2where *a* and *b* are the intercept and slope of the calibration curve, respectively. We obtained an outstanding LOD of 48.7 fg/mL (3.74 fM) with a good linear correlation coefficient of *R*^2^ = 0.991 for PCT detection, which is significantly lower than the clinical cutoff value. More information on the linear dynamic range of the sensor can be found in Supporting Information S5.

Table 1 compares the performance of our WENLISA biosensor with reported optical biosensor technologies for PCT detection.^3,19,25,30−32^ The LOD value obtained by WENLISA is the lowest among existing optical technologies. In addition, PCT detection was rapid and cost-effective. Hence, with a short detection time, low LOD, and low cost, our WENLISA sensor is capable of the ultrasensitive detection of PCT.

**Table 1:** WENLISA Performance Results Compared with Previously Reported Optical Biosensors for PCT Detection^a^

method	LOD (pg/mL)	detection time (min)	cost	ref
NEDPI	21.3	<15	low	3
TIR-FIA	20	9	high	19
FONLISA	0.095	15	medium	25
ICA	3	15		30
SPR	4200	<10	low	31
micromotor	10	5	low	32
WENLISA	0.048	∼8	low	this work

a NEDPI, nanoparticle enhanced digital plasmonic imaging; TIR-FIA, total internal reflection fluorescence immunoassay; FONLISA, fiber optic nanolinked immunosorbent assay; ICA, immunochromatographic assay; SPR, surface plasmon resonance.

To validate the capacity and accuracy of our WENLISA biosensors, three plasma samples provided by Taichung Veterans General Hospital were evaluated. Figure 3a shows the real-time optical response using a WENLISA biosensor for a blood plasma sample from a patient diagnosed with initial systemic infection (sample A). When the PBS solution was injected into the sensor chip, a stable baseline was observed with a low system noise of σ = 1.6 × 10^–4^. Subsequently, upon injection of the diluted plasma sample into the chip, the intensity decreased exponentially as a result of molecular binding during detection because of the selective capture of the analyte by the capture antibody. Next, PBS was again injected into the sensor to wash the unbound AuNP@Ab^D^, leading to a decrease in background signal and increase in transmitted light intensity. Next, we calculated the detected concentration to check the ability of WENLISA for clinical detection. To determine the detected concentration, using the normalized sensor response generated by the blood plasma sample with a value Δ*I*/*I*_0_ = 0.0138, the sample PCT concentration can be calculated using
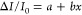
3Thereafter, the sample PCT concentration was determined to be 4.68 ng/mL, which closely aligns with the clinically accepted electrochemiluminescence assay (ECLISA) method. This detected concentration leads to the clinical diagnosis of a positive case for sepsis.

**Figure 3:**
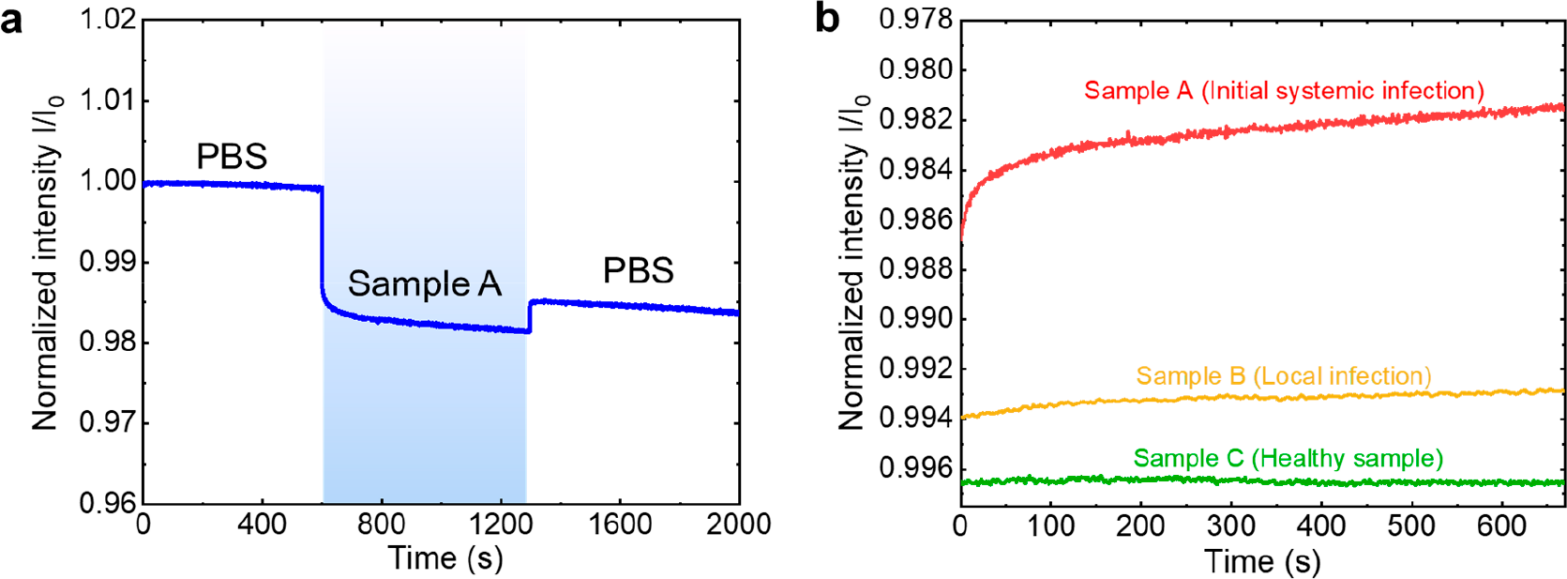
(a) Real time responses for a blood plasma sample from a patient diagnosed with initial systemic infection. (b) Normalized intensity as a function of time for cases of initial systemic infection, local infection case, and healthy individual.

Figure 3b shows the real-time optical responses of WENLISA for blood plasma samples from a healthy case, a local infection case, and an initial systemic infection case. Using our WENLISA biosensor, the PCT concentrations of the samples were determined to be 0.116, 0.132, and 4.680 ng/mL for the healthy case, local infection case, and initial systemic infection case, respectively. Although there are differences in detected concentrations between the WENLISA method and the ECLISA method, the results still reasonably agree on the same order of magnitude as shown in Table 2. The results also demonstrate the capability of WENLISA to discriminate from a negative case. Furthermore, these results clearly demonstrate that our WENLISA biosensor can indeed detect and distinguish PCT in blood plasma samples in ∼8 min for rapid and early sepsis diagnosis. It is noted that experimental results by any technique can have errors due to sampling, operational bias, precision of the method, and preparation of the calibration standard. To reduce these errors, a more rigorous validation plan is needed to standardize the sample preparation procedures and assay protocols. Addressing these factors causing errors and implementing quality control measures to test clinical samples with a much larger sample size may minimize the discrepancy between WENLISA and the ECLISA method. With the record-low LOD for PCT detection, low nonspecific adsorption, short-detection time of ∼8 min, and cost-effective and scalable fabrication, the WENLISA biosensor can enhance clinical management and improve patient outcomes in the context of sepsis diagnosis and treatment.

**Table 2:** Comparison of Sample Concentration Detection by WENLISA and ECLISA

sample	WENLISA (ng/mL)	ECLISA (ng/mL)	remark
A	4.680	1.8	initial systemic infection case
B	0.132	0.35	local infection case
C	0.116	0.047	healthy case

In summary, this study proposes a novel WENLISA optofluidic biosensor for the rapid, ultrasensitive, and low-cost detection of PCT for early sepsis diagnosis. The biosensor was constructed by using rapid, vacuum-free, and lithography-free processes suitable for mass production. Analytical sensitivity was significantly enhanced using a new sensing strategy employing a gold nanoparticle-labeled detection antibody and waveguide-immobilized capture antibody to form a unique sandwich architecture. The biodetection experiments clearly demonstrate the ability of WENLISA to detect PCT with a LOD as low as 48.7 fg/mL (3.74 fM) and short detection time of ∼8 min. By evaluating the detected PCT concentrations, it was verified that the WENLISA biosensor can distinguish between healthy individuals and patients with local infection or initial systemic infection. These results confirm the potential of the WENLISA platform for ultrasensitive detection of crucial biomarkers present in low concentrations, enabling the accurate and prompt diagnosis of different diseases and thus improving clinical outcomes for patients.

## Data Availability

The data that support the findings of this study are available from the corresponding author upon reasonable request.

## Supporting Information Available

Description of biosensor fabrication, optical detection system, simulation methods and analysis for the optical field of waveguide with gold nanoparticles, calculation of background absorption, discussion on the linear dynamic range, chemicals and materials, reagents and materials, preparation and characterization of AuNPs, immobilization of capture antibody, preparation of AuNP@Ab^D^, and preparation of standard and clinical specimens (PDF)
