# Supplementary material for: Ultrasensitive and Rapid Detection of Procalcitonin via Waveguide-Enhanced Nanogold-Linked Immunosorbent Assay for Early Sepsis Diagnosis
Source: Nano Lett. 2024 Jan 22;24(8):2596–602. doi: 10.1021/acs.nanolett.3c04762 (PMC10906069; doi:10.1021/acs.nanolett.3c04762)
Supplement: Supplementary file 1 — nl3c04762_si_001.pdf [file nl3c04762_si_001.pdf]

Supporting Information

Ultrasensitive and rapid detection of Procalcitonin via waveguide-enhanced nanogold-linked immunosorbent assay for early sepsis diagnosis

Devesh Barshilia [a,b], *Jhen-Jie Huang* [c], *Akhil Chandrakanth Komaram* [c], *Yi-Che Chen* [c],

Chun-Da Chen [d], *Min-Yu Syu* [d], *Wen-Cheng Chao* [e], *Lai-Kwan Chau* [c,f,]*, and *Guo-En Chang* [a,b,f,]*

[a]Department of Mechanical Engineering, National Chung Cheng University, Chiayi 621301, Taiwan

[b]Advanced Institute of Manufacturing with High-Tech Innovations (AIM-HI), National Chung Cheng University, Chiayi 621301, Taiwan

[c]Department of Chemistry and Biochemistry, National Chung Cheng University, Chiayi 621301, Taiwan

[d]Department of Laboratory Medicine, National Taiwan University Hospital Yunlin Branch, Yunlin 640, Taiwan

[e]Department of Critical Care Medicine, Taichung Veterans General Hospital, Taichung 402202, Taiwan

[f]Center for Nano Bio-Detection, National Chung Cheng University, Chiayi 621301, Taiwan

*Correspondence Emails: Lai-Kwan Chau: chelkc@ccu.edu.tw

Guo-En Chang: imegec@ccu.edu.tw

S1. Fabrication of WENLISA sensor chips

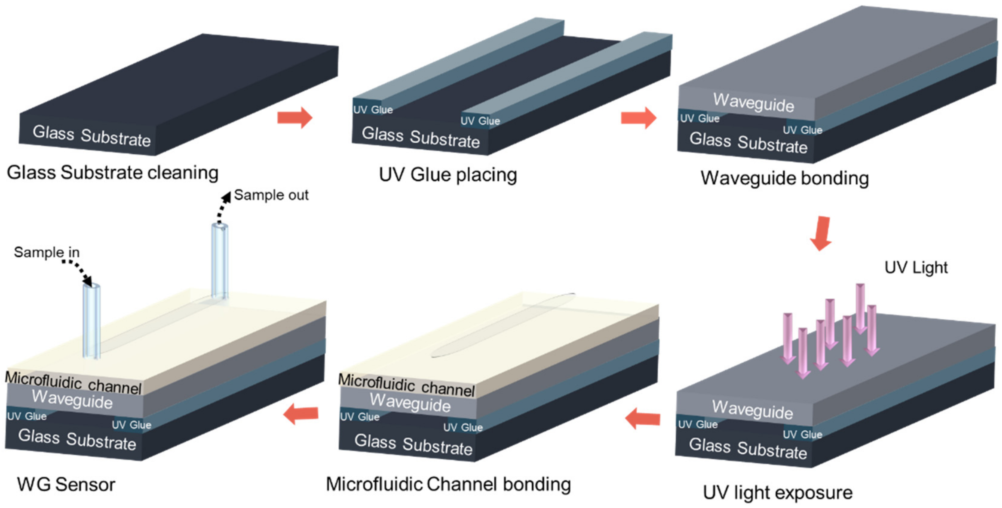

Figure S1: Fabrication flow of the WENLISA biosensor.

The WENLISA biosensors were fabricated without lithography or vacuum techniques. Figure S1 illustrates the fabrication flow of the WENLISA biosensor. First, a glass slide with dimension 25.4 × 52 × 1 mm was used as the substrate. Thereafter, the WG and microfluidic channel were washed with DI water and dried with a nitrogen gun, followed by oxygen plasma treatment for 30 min. Subsequently, the WG and channel were baked in an oven for 1 h. A glass WG (24 × 50 × 0.2 mm) was used as the WG in the sensor, which was cleaned and glued to the opposite side of the substrate, followed by irradiation using a UV lamp for 15 min for glue hardening. An injection molding technique was used to prepare microfluidic channels of dimensions 32 × 3 × 0.2 mm. Finally, flexible tubes were connected to the microfluidic channel to act as the fluid injection inlet and outlet. The fabrication technique employed for the sensor is rapid, simple, and avoids costly lithography processes, thereby producing low-cost (< 1 USD) WENLISA biosensors.

S2. Optical Detection System

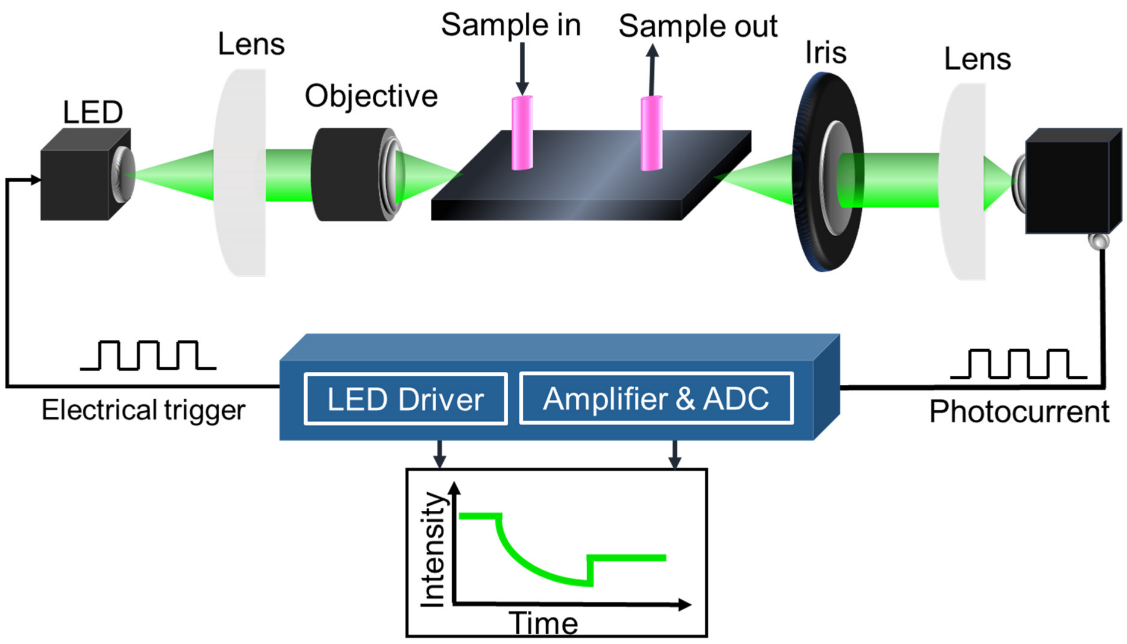

Figure S2: Optical detection system.

Figure S2 shows a schematic of the optical detection system setup for detecting real-time PCT signals from the developed WENLISA biosensor. The measurement system precisely evaluates the change in the output signal upon injection of different PCT concentrations into the sensor. Our optical detection system allows for real-time, simple, and rapid biodetection of PCT biomarkers.

A commercially available low-cost green LED with high stability was employed as a light source in the optical detection system. The homemade driver produces a 1 kHz square wave with a 50 % duty cycle and directly modulates the LED. The output intensity from the source was collimated using a converging lens and focused on the facet of the slab WG using a 20× objective lens. For precise alignment of the WENLISA sensor, the fabricated sensor was aligned on a 3-axis translational stage, and light was coupled to the sensor chip. The intensity

transmitted from the WG output facet was filtered using an adjustable iris, followed by focusing on the Si photodetector to convert output light intensity into a photocurrent. Using an A/D converter, the output current was transformed into digital signals, which were demodulated using a self-developed digital lock-in program.

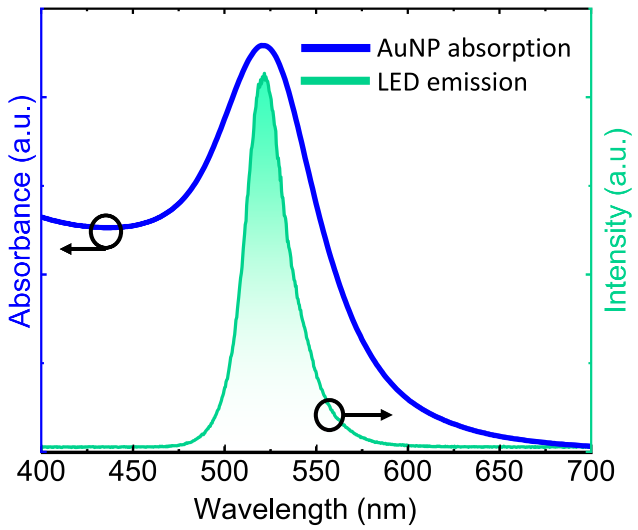

Fig S3: LED emission spectrum and AuNP absorption spectrum.

Figure S3 shows the measured LED spectrum and the absorption spectrum of immobilized AuNPs on a glass substrate. The LED has a peak emission wavelength at 521.5 nm with a full-width-at-half-maximum (FWHM) of 29 nm. Upon immobilization of the AuNPs on a glass substrate and functionalization of the AuNP surface with a self-assembled monolayer, the plasmonic peak typically occurs at 520 nm. Thus, the LED emission peak can match the resonance peak of AuNPs to excite nanoplasmonic resonance.

S3. Electric field distribution

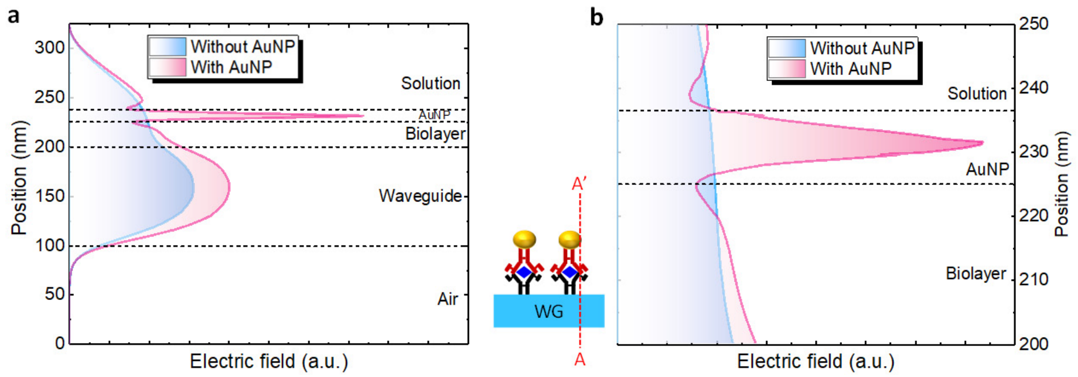

Figure S4: (a) Electric field distributions in the entire WG and its neighboring layers and (b) near the biolayer with and without AuNPs at λ=521.5 nm along the AA' line.

Table S1. Refractive indices of materials at λ=521.5 nm [S1, S2, S3].

**Material**	Waveguide	AuNP	Solution
**Refractive index**	1.52	0.72+ 2.2 i	1.33402

Finite-element-method (FEM) method was conducted to simulate the electric field distribution. A plane wave was employed as the light source which was excited at one facet of the waveguide. A perfect-matching-layer was placed at the other facet of the WG to ensure no light reflection. The refractive indices at λ=521.5 nm for the WG, AuNP, and solution are n_W=1.52, n_{Au}=0.72+ 2.2i, and n_s=1.33402, respectively [S1, S2, S3]. The thickness of the air gap, waveguide, biolayer and the diameter of AuNP applied in the simulation models are 100 nm, 100 nm, 25 nm and 13 nm respectively. The field distributions were then obtained. Figure S4(a) shows the electric field distributions in the WG and its neighboring layers with and without AuNPs along the AA' line. Without AuNPs, the decay length evanescent field is

determined to be 77 nm, which is much larger than the thickness of the biolayer. It clearly shows that in the presence of AuNPs the strong nanoplasmonic resonance occurs which clearly modifies the evanescent wave distribution in comparison to that without AuNPs. To provide a clearer presentation, we have included a zoomed version of the electric field distribution with and without AuNPs in Figure S4(b). This depiction illustrates the modifications in the electric field within the biolayer in the presence and absence of AuNPs. The introduction of the sandwich-like nanocomplex with AuNP linked to the detection antibody induces a strong AuNP resonance mode when the plane wave is incident on the WG. This strong resonance mode remarkably intensifies the absorption in the vicinity of the analyte molecule and hence the optical confinement value of up to 10% in the biolayer. It should be noted that the distribution of the electric field around AuNPs is asymmetrical (as shown in Fig. S4 and Fig. 1(g),), due to the interplay between the incident electromagnetic (EM) field under guided mode propagation in the glass WG and the collective oscillations of conduction electrons in the metal. For a metal nanoparticle above a dielectric substrate, the substrate-induced screening field is nonuniform across the nanoparticle [S4]. Furthermore, the nanoparticle shifts a large part of the near-field EM toward the substrate, which has a higher RI than the surrounding media [S5]. Such a coupling between the nanoparticle and the substrate results in the red shift of the plasmon resonance and also the lowering of the nanoparticle's extinction cross-section [S6]. When the separation distance x between the nanoparticle and the substrate is smaller the diameter D of the nanoparticle, red shift of the plasmon resonance is significant [S7]. On the other hand, when $x >> D$, the effect of mirror induced charges in the substrate becomes negligible. Interestingly, using our WENLISA approach, the mean diameter of AuNPs is about 13 nm while x is about 28 nm, suggesting that the AuNP's extinction cross-section is essentially at its maximum value to yield optimum sensitivity of the biosensor.

S4. Background absorption

To evaluate sensor response considering the background nonspecific adsorption of AuNP@Ab^{D} in a blank, the background adsorption level (*B*)[32] was defined as the mean plus three times the standard deviation of the background nonspecific adsorption of AuNP@Ab^{D}. To prevent false positive signals resulting from background adsorption of AuNP@Ab^{D} on the waveguide surface, the sensor response must exceed the *B* value, which acts as a cut-off value for establishing the practical LOD during calibration and when analyzing actual samples. Our sensor response due to the background adsorption of AuNP@Ab^{D} provided a value of B = 0.0012 using an average sensor response of 0.00082 ± 0.00014 in the non-specific adsorption tests. Hence, any sensor response higher than this cut-off value will be considered as a positive result. In practice, each batch of sensors and the corresponding AuNP@Ab^{D} probes will require independent *B* value calibrations.

S5. Linear dynamic range

The wide linear dynamic range of 4 orders (as shown in Figure 2d) is ascribed to the large surface-to-volume ratio in the microchannel and the ultrahigh sensitivity of the biosensor. At the lower concentration limit (7.7×10^{-15} M) of the calibration curve, assuming most PCT molecules in the microchannel (~20 μL) were captured on the waveguide surface due to the law of mass action, then the number of AuNPs bound on the waveguide surface was in the order of magnitude of 10^5. In other words, the biosensor can offer sufficient signal with only about 10^5 AuNPs bound on the waveguide surface. At the upper concentration limit (7.7×10^{-11} M) of the calibration curve, the number of PCT molecules in the microchannel was on the order of magnitude of 10^9 while the large sensor surface of 96 mm^2 provided the number of capture antibody molecules in the order of magnitude of 10^{11} [S8]. That is to say, PCT molecules at the upper concentration limit is still far before the saturation point and hence the calibration curve is still linear. It should be noted the upper concentration limit could also be restricted by the AuNP@Ab^D concentration.

S6. Chemicals and materials

Hydrogen tetrachloroaurate (III) trihydrate was purchased from Alfa Aesar (USA). Sodium citrate and ethanolamine were purchased from Baker Chemicals. 3-Aminopropyltriethoxysilane (APTES) was obtained from Acros Organics. 16-Mercaptohexadecanoic acid (MHDA), 1-ethyl-3-(dimethylaminopropyl)-carbodiimide hydrochloride (EDC), N-hydroxysuccinimide (NHS), dextran (70 kDa), sodium cyanoborohydride, glutaraldehyde, sodium bicarbonate, bovine serum albumin, and serum were purchased from Sigma-Aldrich. SBSH was obtained from Taiclone (Shanghai, China). Sodium metaperiodate was purchased from Merck. Acetic acid was purchased from Fluka (Tokyo, Japan). Tween 20 and sodium acetate were purchased from Showa (Osaka, Japan). PBS tablets were purchased from BioVision (San Diego, CA, USA). Ethanol was purchased from Echo Chemical Industries (Tokyo, Japan). Methanol was purchased from Aencore Chemicals (Tokyo, Japan). Ultrapure water (18.2 MΩ cm) was produced via a Milli-Q water system (Millipore) to prepare all aqueous solutions. Mouse procalcitonin monoclonal antibody (MBS312689) was used as the capture antibody and procalcitonin monoclonal antibody (MBS310732) was used as the detection antibody; both antibodies were obtained from MyBioSource. Human recombinant PCT (12.8 kDa) was purchased from ProSpec.

S7. Reagents and materials

Hydrogen tetrachloroaurate (III) trihydrate was purchased from Alfa Aesar (USA). Sodium citrate and ethanolamine were purchased from Baker Chemicals. 3-Aminopropyltriethoxysilane (APTES) was obtained from Acros Organics. 16-Mercaptohexadecanoic acid (MHDA), 1-ethyl-3-(dimethylaminopropyl)-carbodiimide hydrochloride (EDC), N-hydroxysuccinimide (NHS), dextran (70 kDa), sodium cyanoborohydride, glutaraldehyde, sodium bicarbonate, bovine serum albumin, and serum were purchased from Sigma-Aldrich. SBSH was obtained from Taiclone (Shanghai, China). Sodium metaperiodate was purchased from Merck. Acetic acid was purchased from Fluka (Tokyo, Japan). Tween 20 and sodium acetate were purchased from Showa (Osaka, Japan). PBS tablets were purchased from BioVision (San Diego, CA, USA). Ethanol was purchased from Echo Chemical Industries (Tokyo, Japan). Methanol was purchased from Aencore Chemicals (Tokyo, Japan). Ultrapure water (18.2 MΩ cm) was produced via a Milli-Q water system (Millipore) and was used to prepare all aqueous solutions.

S8. Preparation and characterization of AuNPs

To prepare the AuNPs, 20 mL aqueous solution of 0.88 mM $HAuCl_4$ was boiled for 10 min with stirring. Subsequently, a 2.4 mL solution of 1% sodium citrate was rapidly added to the $HAuCl_4$ solution with vigorous stirring, and the resulting solution was heated for 20 min continuously. During heating, the color of the solution changed from yellow to ruby-red, confirming the formation of AuNPs. Then, the ruby-red solution was stirred continuously for 20 min and allowed to cool to room temperature. The freshly prepared AuNP solutions were diluted to an absorbance value of 2.0 a.u. and stored in a 4 °C environment as stock solutions until further use.

The AuNP solutions were characterized by UV-vis absorption spectroscopy using a UV-vis spectrophotometer (GBC Cintra 2020), and a peak wavelength of 519 ± 1 nm was obtained. The mean diameter of the AuNPs was determined to be 12.0 ± 1.4 nm through a transmission electron microscope (TEM, JEOL JEM-2010). The mean hydrodynamic diameter of the AuNPs was measured using dynamic light scattering (DLS, Zetasizer Nano ZS90, Malvern) and was estimated to be 18.8 ± 1.0 nm.

S9. Immobilization of capture antibody

For the immobilization of the capture antibody on the WG surface, 2% of 3-aminopropyl triethoxysilane (APTES) in 95% ethanol was used to modify the WG surface for 15 min and functionalize the WG surface with amine groups. The WG surface was then treated with a pre-oxidized 70 KDa dextran solution in 0.1 M acetate buffer (pH 5). This dextran solution was prepared by first mixing dextran (5 μM) with $NaIO_4$ (60 mM) in a 0.1 M acetate buffer for 10 min in the dark and adding $NaBH_3CN$ (1.6 mM). The dextran treatment reaction was carried out for 16 h to produce a dextran layer on the WG surface, which served as an anti-nonspecific adsorption layer. Subsequently, a solution of 0.26 mM glutaraldehyde and 1.6 mm $NaBH_3CN$ in $NaHCO_3$ with a reaction time of 2 h was used to deactivate the unreacted amine groups. To functionalize the dextran-modified WG for the capture antibody, a solution of 10 μg/mL antibody and 1.6 mM $NaBH_3CN$ in PBS was allowed to react with the dextran-modified WG for 2 h. Subsequently, a solution of 10^{-3} g/mL bovine serum albumin (BSA) in 10 mM PBS as a surface blocker was added to the antibody-conjugated WG surface for 30 min. Finally, a solution of 1M ethanolamine and 1.6 mM $NaBH_3CN$ in ultrapure water was used to react with the resulting WG surface for 10 min to remove the remaining unreacted aldehyde groups. The resulting sensor WG was stored in the dark at 4 °C.

S10. Preparation of AuNP@Ab^D

The detailed procedures for synthesizing the AuNPs are described in Supplementary Materials S8. The AuNP-labelled detection antibody solution was prepared according to a previous report[25]. Briefly, 2 mL aqueous AuNP solution and 2 mL 0.2% Tween 20 solution in ultrapure water were mixed, wherein Tween 20 was used as a stabilizer for the AuNPs. After 1 h, a mixture of 240 μL 0.5 mM sulfobetaine thiol (SBSH) and 60 μL 0.5 mM 16-mercaptohexadecanoic acid (MHDA) was added, and the resulting solution was left undisturbed to react for 16 h and subsequently centrifuged for 20 min. Next, a 100 μL mixture of 10^{-2} M 1-ethyl-3-(3-dimethylaminopropyl)-carbodiimine hydrochloride (EDC), 0.017 M N-hydroxy-succinimide (NHS) and 2 mL 0.1% tween 20 in ultrapure water was added and allowed to react for 30 min to activate the carboxyl group. Subsequently, the unreacted EDC and NHS were removed by centrifugation for 15 min at 10,000 rpm. Finally, a 2 mL solution of PCT antibody (5×10^{-7} g/mL) in PBS was added and allowed to react for 2 h. Next, a 100 μL solution of 0.001 g/mL BSA in PBS was added and left for 30 min and then centrifuged for 20 min at 10,000 rpm. The resulting solution was stored in the dark at 4 ^{o}C. The AuNP@Ab^D product was characterized by UV-vis spectroscopy and dynamic light scattering (DLS). The UV-vis spectra of the AuNP@Ab^D solutions had a peak wavelength of approximately 528 nm, which was red-shifted from the 519 nm peak of the AuNP solution. The mean hydrodynamic diameter of AuNP@Ab^D was 61.9 ± 1.0 nm, which is significantly larger than that of citrate-capped AuNPs at 18.8 ± 1.0 nm.

S11. Preparation of standards and clinical specimens

Standard solutions of PCT at concentrations of 0.10, 1.00, 10.0, 100 pg/mL, and 1.00 ng/mL were prepared in 10 mM PBS at pH 7.4. Blood plasma samples were collected from Taichung Veterans General Hospital. This study was reviewed and approved by the institutional review Broad (IRB) of Taichung Veterans General Hospital (IRB number: SE17139A-3). Written informed consent was obtained from all participants. Sample concentrations were determined using our biosensor and the standard clinical laboratory ECLISA method. Blood plasma samples were diluted by a factor of 2000 in PBS at pH 7.4.

References

S1. Shi, Y. Z., Xiong, S., Chin, L. K., Zhang, J. B., Ser, W., Wu, J. H., Chen, T. N., Yang, Z. C., Hao, Y. L., Liu, A. Q., Determination of size and refractive index of single gold nanoparticles using an optofluidic chip, *AIP Advances* **2017**, *7*, 095024.

S2. Barshilia, D, Chau, .L.-K., Chang, G.-E., Low-cost planar waveguide-based optofluidic sensor for real-time refractive index sensing, *Opt. Express* **2020**, *28*, 27337-27345.

S3. Scaffardi, B, Pellegri, Sanctis, de. O, Tocho, J.O., Sizing gold nanoparticles by optical extinction spectroscopy, *Nanotechnol,* **2005**, *16*, 158-163.

S4. Knight, M. K.; Wu, Y.; Lassiter, J. B.; Nordlander, P.; Halas, N. J., Substrates matter: influence of an adjacent dielectric on an individual plasmonic nanoparticle. *Nano Lett.* **2009**, *9*, 2188-2192.

S5. Martinsson, E.; Otte, M. A.; Shahjamali, M. M.; Sepulveda, B.; Aili, D., Substrate effect on the refractive index sensitivity of silver nanoparticles. *J. Phys. Chem. C* **2014**, *118*, 24680-24687.

S6. Xu, H.; Kail, M., Modeleing the optical response of nanoparticle-based surface plasmon resonance sensors. *Sens. Actuat. B* **2002**, *87*, 244-249.

S7. Pinchuk, A.; Hilger, A.; von Plessen, G.; Kreibig, U., Substrate effect on the optical response of silver nanoparticles. *Nanotechnol.* **2004**, *15*, 1890-1896.

S8. Chaudhari, P. P.; Chau, L.-K.; Tseng, Y.-T.; Huang, C.-J.; Chen, Y.-L., A fiber optic nanoplasmonic biosensor for the sensitive detection of ampicillin and its analogs. *Microchimica Acta* **2020,** *187*, 396.
